# Blueberry supplementation reduces the blood lactate response to running in normobaric hypoxia but has no effect on performance in recreational runners

**DOI:** 10.1186/s12970-021-00423-7

**Published:** 2021-03-30

**Authors:** Jason P. Brandenburg, Luisa V. Giles

**Affiliations:** Department of Kinesiology, School of Kinesiology, University of the Fraser Valley, 45190 Caen Avenue, BC V2R 0N3 Chilliwack, Canada

**Keywords:** Polyphenols, Anthocyanins, Running, Endurance, Altitude, Nutrition

## Abstract

**Background:**

Blueberries are concentrated with anthocyanins possessing antioxidant properties. As these properties counter fatigue, blueberry supplementation may improve performance and recovery, particularly in hypoxia, where oxidative stress is elevated.

**Methods:**

This study examined the effects of blueberry supplementation on running performance, physiological responses, and recovery in normobaric hypoxia. Eleven experienced runners completed a 30-minute time-trial (TT) in normobaric hypoxia (%O_2_ = 15.5 %) on separate days after supplementation with four days of blueberries (BLU) or four days of placebo (PLA). Heart rate (HR), oxygen saturation (SaO_2_) and ratings of perceived exertion (RPE) were monitored during the TT. Blood lactate and fraction of exhaled nitric oxide (F_ENO_) were assessed pre-TT, post-TT, and during recovery.

**Results:**

No significant differences were observed in the distance run during the TT, HR, SaO_2_, and RPE. The post-TT increase in blood lactate was significantly lower in BLU than PLA (*p* = 0.036). Pre-TT and post-TT F_ENO_ did not differ between conditions. Blood lactate recovery following the TT was similar between conditions.

**Conclusions:**

Four days of blueberry supplementation did not alter running performance or cardiovascular and perceptual responses in normobaric hypoxia. Supplementation lowered the blood lactate response to running, however, the significance of this finding is uncertain given the absence of an ergogenic effect.

## Introduction

The elevated metabolic requirements of exercise accelerate the production of reactive oxygen and nitrogen species (RONS) [1]. In small quantities, RONS function as signaling molecules that aid force production, help regulate muscle blood flow, and even trigger adaptations to exercise[2, 3]. However, when the accumulation of RONS is in excess, oxidative stress occurs, and this may contribute to fatigue[3]. The contribution of oxidative stress to the development of fatigue is multifactorial and includes a reduced availability of nitric oxide (NO), which limits the capacity for vasodilation and blood flow[3]. Oxidative stress also disrupts calcium handling and sensitivity, consequently impairing force production[3]. The accumulation of RONS is influenced by the intensity and duration of exercise, as well as the environmental conditions in which the exercise is performed[4]. For example, when exercise is conducted in a hypoxic environment the degree of oxidative stress is heightened and is further implicated in the development of fatigue and decrements in performance[5, 6].

Considering the involvement of oxidative stress in exercise-induced fatigue and that higher levels of oxidative stress occur during exercise performed in hypoxia, strengthening an individual’s antioxidant defenses, thereby preventing an excessive accumulation of RONS, may have an ergogenic effect on performance. Nutritionally, supplementation with polyphenol-rich fruits is one way to enhance an individual’s antioxidant defenses [7, 8]. Polyphenols represent a class of compounds that have anti-inflammatory and antioxidant properties. Polyphenols are typically found in fruits and vegetables, and blueberries are considered to be an abundant source of polyphenols[9, 10]. Within blueberries, polyphenol compounds include phenolic acids and flavonoids, the most notable of which are anthocyanins[11]. From a health perspective, supplementation with blueberries has cardiovascular benefits including improved blood pressure, endothelial function, and arterial stiffness[12]. With respect to its ergogenic potential, acute blueberry supplementation mitigated the oxidative stress response to prolonged running[13]. However, as running performance was not assessed, it is unknown if this benefit translates into improved performance. Recent research suggests that although acute blueberry supplementation did not improve 8-km running time-trial performance in normoxia, it did lessen the metabolic disturbances to running[14]. If blueberry supplementation lessens the metabolic disturbances to exercise in normoxia, it seems plausible that supplementation could aid performance in an environment in which the metabolic and oxidative stress disturbances to exercise are accentuated, as would be the case in hypoxia.

Currently, the ergogenic effect of acute supplementation with polyphenol-rich fruits on endurance performance is unclear due to mixed findings [15]. Moreover, the effects of anthocyanin-rich fruit supplementation (and specifically blueberries), on endurance performance and the physiological responses to exercise in hypoxia are not well understood due to the paucity of research. Consequently, the aim of the present study was to explore the influence of acute blueberry supplementation on 30-minute running time-trial performance and the physiological responses to running in normobaric hypoxia.

## Materials and Methods

### Study Design

This study used a randomized, double-blind, placebo-controlled, crossover design with a 14-day washout period between experimental sessions. Participants visited the laboratory on three occasions. In the familiarization visit, participants performed the 30-minute treadmill time trial (TT) in normobaric hypoxia (F_i_O_2_=15.5 %) and a graded running test to volitional exhaustion in normoxia to determine maximal oxygen consumption (VO_2max_). The last two visits served as experimental sessions. During each experimental session, runners completed a 30-minute TT in normobaric hypoxia. Heart rate (HR), rating of perceived exertion (RPE), and oxygen saturation (SaO_2_) were monitored throughout the TT. Blood lactate was assessed prior to, as well as 5, 15 and 30 min following the TT. Additionally, saliva and fraction of exhaled nitric oxide (F_ENO_) were sampled pre- and post-TT. In the four days before each experimental session, participants supplemented with 4 days of blueberry powder (BLU) or 4 days of a placebo powder (PLA). For each participant, sessions were conducted at the same time of day (± 30 min).

### Subjects

Eleven recreational runners (4 males; 7 females) volunteered for this study (mean ± SD: age = 28.4 ± 7.5 years; height = 170.9 ± 9.0 cm; body mass = 65.6 ± 9.7 kg; VO_2max_ = 49.9 ± 9.3 mL·kg^− 1^·min^− 1^). All participants were non-smokers; free from cardiovascular, respiratory, and oral/gum disease; and free from musculoskeletal injury. None of the participants had been exposed to altitude (natural or simulated equivalent to ≥ 1200 m above sea level) within the 3 months prior to the start of the study. 

### Graded running test and VO_2max_

During the familiarization session, the graded running test began at a treadmill speed considered comfortable by the participant (Alpine Runner, True Fitness Technology, St. Louis, MO). Treadmill gradient was increased by 2 % every 2 min for the first 6 min of the test and then by 1 % every 1 min until volitional exhaustion. Oxygen consumption was measured continuously throughout the test using a gas analysis system (TrueOne, Parvomedics, Sandy, Utah). VO_2max_ was determined as the highest 30-s average. All participants performed a 5-minute warm-up at a self-selected speed before commencing the graded running test.

### Experimental Protocols

Prior to the start of each experimental session, participants rinsed their mouth with distilled water and were fitted with a HR sensor and chest strap (Polar H7, Kempele, Finland). Participants were then moved into the altitude chamber (Altitudetech Inc, Kingston, ON, Canada) where they rested in a seated position for 10 min. During this time each participant’s food/activity log was reviewed to ensure compliance with study requirements.

Afterwards, participants provided saliva samples and pre-TT measures of F_ENO_ and blood lactate were administered. Blood lactate was determined using a finger prick and handheld lactate analyzer (Lactate Pro; Arkray KDK Corp., Kyoto, Japan). The Lactate Pro analyzer, when compared to laboratory-based analyzers, has been shown to be both reliable (Coefficient of variation [CV] ranging from 2.7 to 5.7 %) and valid (SEE ranging from 0.5 to 1.1; Bias raging from − 0.6 - -1.7) [16–18]. F_ENO_ was assessed using a handheld electrochemical analyzer (NIOX MINO, Aerocrine AB, Solna, Sweden) following previously established guidelines[19]. Once the pre-TT measures were completed, participants completed a 5-minute self-paced warm-up followed by the 30-minute TT; both of which were performed on a non-motorized treadmill (Trueform Runner; Model: Performance; Connecticut, USA). Runners were instructed to run as far as possible during the 30 min (i.e. best effort). Runners were blinded to distance and running speed but were aware of the elapsed time. A fan was placed in front of the participant to simulate outdoor running conditions. A non-motorized treadmill was utilized for the TT because changes in running speed (i.e. pacing) are instantaneous, influenced by subconscious fatigue, and characteristic of over-ground running[20]. In experienced runners, like those in the present study, a TT on non-motorized treadmill has been shown to be reliable after a single familiarization session[16]. HR and SaO_2_, by fingertip pulse oximeter (MightySat, Masimo Corp, Irvine, CA), were recorded throughout the TT while RPE (Borg 6–20 scale) was recorded every 5 min.

Upon completion of the 30-minute TT, saliva was sampled, F_ENO_ was assessed at 4 min post-TT, and blood lactate measurements were repeated at 5, 15, and 30 min post-TT. To assess lactate recovery, all post-TT values were normalized to the 5-minute post-TT value[21].

### Salivary Analyses

Saliva was collected to measure cortisol, uric acid (UA), interleukin-6 (IL-6), and C-reactive protein (CRP). Salivary biomarkers were measured due to the ease and non-invasive nature of sample collection. The biomarkers analyzed in the present study have been shown to be valid and reliable indicators of the physiological stress to running[22, 23]. At each of the two saliva collection time points, two ml of saliva were collected using the passive drool method. Following saliva collection, samples were frozen at -20 °C until analyses were conducted. Saliva samples were assayed using commercially available highly sensitive enzyme linked immunoassay kits (Salimetrics, PA, USA). Samples were analyzed in duplicate. The intra-assay CVs for the above-mentioned saliva assays were below 5 %.

### Supplementation and Diet/Physical Activity Restrictions

In the four days leading up to each experimental session, runners ingested either freeze-dried blueberry powder (BLU) (50/50 blend of Vaccinium virgatum/Vaccinium corymbosum, Oxygen Radical Absorbance Capacity [ORAC] = 831 µmole TE/g, Table 1) or placebo powder (PLA). PLA, manufactured to have a similar taste and appearance to the blueberry powder, was made from maltodextrin, fructose, dextrose, citric acid, malic acid, silicon dioxide (flow agent), xanthan gum, artificial colors, and artificial and natural flavours.

**Table 1:** Nutrient composition of the freeze-dried blueberry and placebo powders

	Freeze-dried blueberry powder (per 24 g packet)	Placebo powder (per 24 g packet)
Energy (kcal)	96	94
Total Carbohydrates (g)	21.8	22.7
Protein (g)	1	0.19
Fat (g)	0.6	0.02
Dietary Fiber (g)	5.1	0
Vitamin C (mg)	3.9	0
Phenolics (mg)	811	0
Anthocyanins (mg)	336	0

During the supplementation period, participants ingested 3, 24 g packets of blueberry or placebo powder per day (BLU = ~ 500 g of fresh blueberries/day). Ingestion of the packets was distributed evenly throughout the day with the final packet ingested 2 h prior to the start of the session. For ingestion, the powder was mixed with about 250 ml of water. The antioxidant and anthocyanin content of the blueberry powder used in the present study (Table 1) was assessed in the batch by a commercial laboratory (International Chemistry Testing, Milford, MA). Antioxidant activity was assessed using the Oxygen Radical Absorbance Capacity (ORAC) test based on the methods of Ou et al.[24]. The anthocyanin assay was based on the methods described by Lee et al.[25].

During each 4-day supplementation period participants were asked to limit the intake of other foods/drinks high in polyphenols as well as refrain from taking other supplements and anti-inflammatory medications. Additionally, participants avoided caffeine and alcohol intake 8 and 24 h (respectively) prior to the start of each session. Participants also abstained from moderate to vigorous intensity physical activity within 24 h of each session. To facilitate this, participants completed a 4-day food and physical activity diary before the first experimental session and were instructed to replicate this diary before the second experimental session. 

### Statistical analysis

Normality of the data was evaluated using the Shapiro–Wilk test. Mauchly test of sphericity was performed to assess the homogeneity of data. Where violations were present, Greenhouse–Geisser adjustments were made. Between-supplement differences in 30-min running distance, average HR, maximum HR, average SaO_2_, RPE, pre-TT salivary biomarkers, and pre- to post-TT change in salivary biomarkers were analyzed using a paired t-test. Differences in F_ENO_ and blood lactate were analyzed using a repeated measures ANOVA with 2 supplement conditions by 2 time points (1 pre- and 1 post-TT measure). Differences in blood lactate recovery were analyzed using a repeated measures ANOVA with 2 supplement conditions by 3 time points (5-min, 15-min, and 30-min post-TT measures). Post hoc analysis was performed using t-tests with a Bonferroni correction. Statistical significance was set at *p* < 0.05. To estimate the magnitude of the effects from supplementation, Cohen’s d effect sizes (ES) were calculated with the magnitude of effects considered small (0.20–0.49), moderate (0.50–0.79) and large (> 0.80). Additionally, Pearson correlation coefficients were used to explore relationships between the study variables. Data are presented as mean ± SD. All statistical analyses were conducted using SPSS v 20.0 (SPSS Inc., Chicago, IL, USA).

## Results

No difference in the distance achieved during the 30-minute TT in normobaric hypoxia was observed between supplement conditions (Table 2). Heart rate, SaO_2_, and RPE responses during the TT were not different between the two supplement conditions (Table 2).

**Table 2:** Performance and physiological variables during the 30-minute time trial completed in normobaric hypoxia

Variable	PLA	BLU
**30-minute time trial** (km)		
0–15 min split	2.21 ± 0.31	2.22 ± 0.38
15–30 min split	2.26 ± 0.32	2.24 ± 0.33
Total distance	4.46 ± 0.62	4.47 ± 0.69
**Heart Rate** (HR)		
Average HR during TT	176.3 ± 13.7	175.3 ± 12.7
Maximum HR during TT	187.3 ± 15.1	186.6 ± 13.4
**%SaO**_**2**_		
30-minute average during TT	89.3 ± 1.4	89.2 ± 2.1
**Rating of Perceived Exertion** (RPE)		
Maximum RPE during TT	18.1 ± 1.4	18.5 ± 1.2

**Fig. 1:**
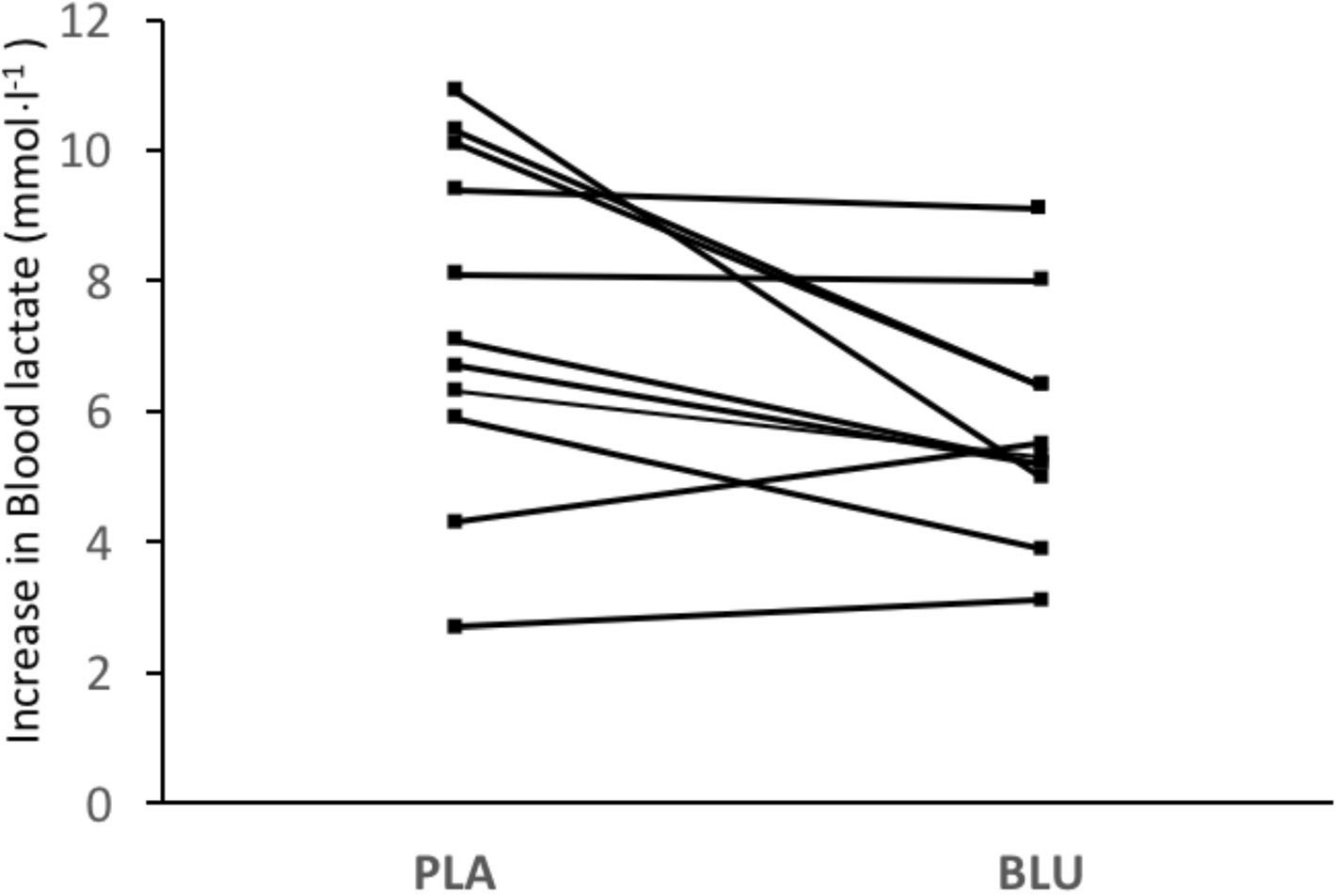
Individual increases in blood lactate concentrations (pre-TT to 5-minute post-TT) in response to PLA and BLU

Pre- and post-TT F_ENO_ levels were not different between the two supplement conditions (Table 3). In both conditions, blood lactate significantly increased following the 30-min TT (*p* < 0.001) with the increase in PLA being significantly greater than BLU (*p* = 0.036) (Table 3). Post-hoc comparisons indicated the blood lactate concentration following the TT was significantly lower in BLU than PLA (*p* = 0.02). A Cohen’s d showed a moderate effect size (*d =* 0.76) for the attenuated post-TT increase in blood lactate following BLU. Additionally, 8 of the 11 runners experienced less of an increase in blood lactate in response to BLU (Fig. 1). There was no significant difference in blood lactate recovery at 15 min as well as 30 min post-TT, although a moderate effect size was observed at 30 min (*d* = 0.51) (Table 3).

**Table 3:** Blood lactate, F_ENO_, and salivary biomarker results before and after the 30-minute time trial

Variable	PLA	BLU
**Exhaled Nitric Oxide** (ppb)		
Pre-exercise	18.5 ± 8.2	18.7 ± 6.1
Post-exercise	17.2 ± 6.4	17.2 ± 4.2
**Blood Lactate** (mmol⋅l^− 1^)		
Pre-TT	1.5 ± 0.4	1.5 ± 0.4
5-minute post-TT	8.9 ± 2.7	7.2 ± 1.9*
**Blood Lactate Recovery** (% of 5-min post-TT)		
Recovery at 15 min post-TT	57.5 ± 16.7	56.7 ± 9.8
Recovery at 30 min post-TT	37.2 ± 12.1	32.5 ± 5.1
**Salivary Cortisol** (µg/dL)		
Pre-TT	0.23 ± 0.11	0.16 ± 0.06
Post-TT	0.53 ± 0.34	0.52 ± 0.27
**Salivary Uric Acid** (mg/dL)		
Pre-TT	3.11 ± 0.81	2.72 ± 0.89
Post-TT	3.04 ± 1.26	2.83 ± 0.93
**Salivary IL-6** (pg/mL)		
Pre-TT	8.9 ± 6.4	7.4 ± 4.2
Post-TT	23.2 ± 9.2	24.3 ± 19.8
**Salivary CRP** (pg/dL)		
Pre-TT	6162 ± 5547	6504 ± 8389
Post-TT	14,796 ± 20,641	10,771 ± 13,987

* = Significantly different than PLA at same time point (*p* = 0.02)

No significant pre-TT differences were observed in any of the salivary biomarkers between PLA and BLU (Table 3). However, a Cohen’s d showed a moderate effect size (*d =* 0.68) for lower pre-exercise cortisol levels in response to BLU. The pre- to post-TT increases in cortisol and IL-6 were significant (*p* < 0.001 and *p* = 0.005, respectively) with no differences between the two supplement conditions. For UA and CRP, pre- to post-TT changes were not significant.

A moderate negative relationship was observed between the PLA vs. BLU difference in pre-TT F_ENO_ and the PLA vs. BLU difference in the pre-TT to post-TT increase in blood lactate concentration (r=-0.50, *p* = 0.069).

## Discussion

The present study examined the effect of acute blueberry supplementation on running performance and physiological responses in normobaric hypoxia. Four days of supplementation had no influence on 30-minute TT performance. However, although most of the physiological variables remained unaffected following supplementation, the post-TT increase in blood lactate was attenuated following blueberry supplementation.

Following the 30-minute TT, blood lactate was 1.7 mmol⋅l^− 1^ or ~ 15 % lower in BLU than PLA (Table 3). When compared to the CV associated with the Lactate Pro analyzer (2.7–5.7 %) [16–18], the 15 % reduction in the blood lactate response points to an intervention effect due to acute blueberry supplementation. This interpretation is reinforced considering 8 of the 11 participants experienced a mitigated post-TT increase in blood lactate following BLU (Fig. 1). The attenuated blood lactate response to the 30-minute TT following BLU is similar to that observed following an 8-km running TT in normoxia in response to a 4-day blueberry supplementation protocol[14]. These findings also mirror the 4 % reduction (not significant) in blood lactate levels in highly-trained cyclists following completion of a TT to exhaustion in normobaric hypoxia (F_i_O_2_ = ~ 17 %) after supplementation with pomegranate extract[26]. In contrast, blood lactate responses to low and moderate intensity cycling in normobaric hypoxia (F_i_O_2_ = ~ 15 %) were not altered after 7 days of supplementation with black currant extract[27]. The contradiction between these findings and those of the present study could be due to differences between the supplementation protocols. In the current study, participants received 336 mg of anthocyanins at each point of ingestion (1008 mg/day); with the final dose ingested 2 h prior to testing. In the study by Willems et al.[28], participants received 105 mg of anthocyanins at each intake (210 mg/day); with the timing of the final supplement greater than 2 h before testing. Physiologically, both the dose of anthocyanins and timing of the last dose influence endothelial function; thought to be principal mechanism underlying the ergogenic effect of anthocyanin-rich fruit supplementation [15]. With respect to dose, acute improvements in endothelial function have been shown to linearly increase in response to an increasing dose of anthocyanins (up to 310 mg)[9]. Further, increases in endothelial function following anthocyanin-rich fruit supplementation peak 1–2 h post-ingestion[9]. With these factors in mind, it seems plausible the greater dose of anthocyanins used in the current study combined with the timing of the last dose relative to exercise (compared to those used by Willems et al.) could have yielded greater improvements endothelial function. If so, improvements in endothelial function may have resulted in increased muscle blood flow, subsequently improving lactate clearance and accounting for the lower post-TT blood lactate values following BLU.

Accounting for the improvements in endothelial function following blueberry supplementation is an increase in NO bioavailability [9]. In the current study, F_ENO_ was used as an indirect marker of NO availability. To this end, significant increases in F_ENO_ have been observed In response to acute beetroot juice supplementation [24, 25]. Accompanying the increases in F_ENO_ were elevated levels of plasma nitrite and nitrate (precursors of NO production); thus suggestive of elevated NO availability in the blood[29]. Considering acute blueberry supplementation also increases NO bioavailability [9], albeit not by the same mechanisms, it was thought that blueberries would have a similar effect on F_ENO_ as nitrate-containing supplements, such as concentrated beetroot juice. However, four days of blueberry supplementation had no effect on F_ENO_ as evidenced by the similar pre-TT values. Consequently, it is uncertain if blueberry supplementation had any impact on NO availability, raising questions about the contribution of altered blood flow to the lower blood lactate response. Despite this, there was a moderate negative relationship (r= -0.50, *p* = 0.06) between the BLU vs. PLA difference in pre-TT F_ENO_ and the BLU vs. PLA difference in the pre-TT to post-TT increase in blood lactate. This suggests that those runners who experienced increases in F_ENO_ in response to blueberry supplementation (in comparison to PLA) tended to experience less of an increase in blood lactate following the TT. Thus, as with other forms of polyphenol-rich supplementation, this relationship indicates the physiological response to blueberry supplementation seems to be individualized (i.e. responders and non-responders)[29]. Two factors thought to contribute to the individual responses to supplementation are training status and diet (i.e. dietary intake of large amounts of polyphenols)[29, 30]. In the context of the present study, the recreational status of the participants along with the restriction of other polyphenol-rich foods/drinks during the 4-day supplementation periods may have contributed to the uniform blood lactate response to the 30-minute TT (i.e. 8 of 11 participants experienced a smaller increase). Although consistent, any ergogenic benefits from this response are uncertain as there were no improvements in 30-minute TT performance.

The absence of any performance advantage following blueberry supplementation is in agreement with recent findings. In a simulated altitude comparable to that of the present study, 10-km treadmill TT performance in trained runners was not significantly altered following a single dose of concentrated beetroot juice (F_i_O_2_=15.4 %)[30]. Similarly, after seven days of blackcurrant extract supplementation recreational cyclists experienced no improvement in a 16.1 km cycling TT in normobaric hypoxia (F_i_O_2_ = ~ 15 %)[27]. When considered collectively, the lack of any significant performance improvement in these studies, and in the present study, may be due to the intensity of exercise task and/or the degree of hypoxia. With respect to intensity, when the duration of the running task was reduced to 1500 m (and therefore intensity increased) supplementation with concentrated beetroot juice significantly improved performance in normobaric hypoxia (F_i_O_2_ = ~ 15 %)[29]. Furthermore, significant improvements in 1500 m running time, but not 10 km running time, were observed at sea level following supplementation with concentrated beetroot juice[31]. The completion of higher intensity exercise, like a 1500 m running TT when compared to the 30-minute TT of the present study or even a 16.1 km cycling TT, is more limited by the delivery of O_2_ to sustain the elevated metabolic requirements of the exercising muscle[2]. Consequently, higher intensity exercise is more likely to benefit following a supplement that improves muscle blood flow via an increase in NO. In the current study, it seems reasonable to suggest the lack of any ergogenic effect following blueberry supplementation may have been due to the relatively modest intensity of the 30-minute TT and that the performance of it was not limited by O_2_ delivery.

## Conclusions

To conclude, four days of blueberry supplementation attenuated the increase in blood lactate to a 30-minute running time trial in normobaric hypoxia. Despite this, there was no improvement in running performance. Consequently, the use of acute blueberry supplementation as an ergogenic aid for endurance performance in hypoxia is not supported.

## Data Availability

The datasets generated and/or analyzed during the current study are not publicly available but are available from the corresponding author on reasonable request.

## Abbreviations

BLU: Blueberry supplementation condition
PLA: Placebo supplementation condition
TT: Time-trial
HR: Heart Rate
RPE: Rating of Perceived Exertion
SaO_2_: O2 saturation
F_ENO_: fraction of exhaled nitric oxide
F_i_**O**_2_: Fraction of inspired O_2_
VO_2max_: Maximal O_2_ consumption
UA: Uric acid
IL-6: Interleukin-6
CRP: C-reactive protein
ORAC: O_2_ Radical Absorbance Capacity
CV: Coefficient of Variation
